# A Lightweight Certificateless Identity Authentication Protocol Using SM2 Algorithm and Self-Secured PUF for IoT

**DOI:** 10.3390/s26092640

**Published:** 2026-04-24

**Authors:** Meili Zhang, Qianqian Zhao, Chao Li, Weidong Fang, Zhong Tong

**Affiliations:** 1 Hubei Provincial Research Institute of Water Resources and Hydropower, Wuhan 430070, China; m18971256899_1@163.com (M.Z.); yuyang202601@126.com (C.L.); tz_hbwri@163.com (Z.T.); 2 Shuiyan Microsystem and Information Technology Co., Ltd., Nanchang 330029, China; 3 Key Laboratory of Science and Technology on Micro-System, Shanghai Institute of Micro-System and Information Technology, Chinese Academy of Sciences, Shanghai 201800, China; weidong.fang@mail.sim.ac.cn

**Keywords:** Internet of Things (IoT), identity authentication, certificateless public key mechanisms based on the SM2 algorithms, physically unclonable functions (PUF)

## Abstract

The rapid proliferation of the Internet of Things (IoT) leaves terminal devices vulnerable to considerable security challenges, notably the absence of robust yet efficient identity authentication mechanisms. Traditional certificate-based approaches incur substantial management overhead and storage expenditure, whereas Identity-Based Cryptography poses inherent key escrow risks. To tackle these challenges, this paper proposes a PUF and SM2-based certificateless identity authentication mechanism that integrates SM2 Certificateless Public Key Cryptography (a Chinese national cryptographic standard) with Physical Unclonable Functions (PUFs). Initially, the proposed solution utilizes PUF technology to derive a unique hardware-generated “fingerprint” from an IoT device, which functions as a root key to generate a partial user private key. This approach essentially binds the terminal’s identity to its physical hardware, thereby effectively mitigating physical cloning attacks against nodes. Moreover, through the adoption of a Certificateless Public Key Cryptography (CLPKC) framework, the complete user private key is jointly generated by a semi-trusted Key Generation Centre (KGC) and the terminal device itself. The comprehensive security analysis proves that the proposed scheme is provably secure under the random oracle model, capable of resisting various common attacks such as physical cloning, man-in-the-middle, and replay attacks. Performance evaluation confirms that the implemented PUF + SM2 certificateless mechanism significantly reduces the size of user public key identifiers to within 64 bytes, offering a substantial advantage over the 1–2 KB certificates typically required in conventional PKI/CA systems, thereby enhancing efficiency in storage and communication.

## 1. Introduction

With the rapid advancement of fifth-generation mobile communication technology (5G), artificial intelligence (AI), and edge computing, the Internet of Things (IoT) has witnessed unprecedented proliferation and widespread adoption, with its applications deeply embedded in critical domains such as smart cities, the Industrial Internet, smart homes, and the Internet of Vehicles [1,2]. The massive scale of IoT terminal devices not only collects data and bridges the physical and digital worlds but also significantly expands the attack surface of cyberspace. Typically deployed in open, unattended, and physically uncontrolled environments, IoT terminals face multiple security threats, including device impersonation, firmware cloning, man-in-the-middle attacks, and sensitive data theft [3]. Consequently, achieving high-strength, lightweight, and verifiable identity authentication for terminal devices in a large-scale, heterogeneous, and resource-constrained IoT setting has become a core bottleneck and key challenge hindering the secure and widespread application of IoT technology [4,5].

The traditional Public Key Infrastructure (PKI)-based digital certificate scheme offers a high level of security. However, the processes involved in certificate issuance, storage, transmission, and verification introduce substantial communication and computational overhead. This makes it difficult to accommodate the typical constraints of IoT terminals, such as limited computing power, small storage capacity, and restricted battery energy. Furthermore, certificate management entails complex lifecycle maintenance—including updates, revocation, and persistent storage—which imposes significant deployment and operational burdens in large-scale IoT systems [6,7]. To simplify key management, Identity-Based Cryptography (IBC) has been proposed by researchers. Nevertheless, its inherent key escrow issue grants the Private Key Generator (PKG) the capability to generate all private user keys. This introduces risks such as a single point of failure and potential insider threats, thereby failing to meet the stringent privacy and security requirements of many practical application scenarios [8,9,10].

Certificateless Public Key Cryptography (CL-PKC) has been introduced, which effectively eliminates the need for digital certificates employed in traditional PKI while also resolving the key escrow issue inherent in Identity-Based Cryptography (IBC) [11,12]. In this system, a user’s private key is composed of two distinct components: One part is generated by the Key Generation Center (KGC), and the other is kept secret and generated independently by the user. This design ensures that the KGC cannot obtain the complete user private key, thereby achieving secure key management without compromising system efficiency [13,14].

With the continuous improvement of China’s requirements for network security and autonomous control, a series of commercial cryptographic algorithm standards (SM2, SM3, SM4, etc.) issued by the State Encryption Administration are increasingly widely used in the Internet of Things, Industrial Internet, and other fields. In the field of IoT authentication, there have been many studies dedicated to optimizing and implementing national security algorithms. For example, Wang et al. designed a lightweight IoT device identity authentication scheme based on SM2 [13], which reduces the computational cost of terminals by optimizing scalar multiplication calculation. Zhang et al. proposed a hybrid authentication protocol that combines SM2 and SM3 [14], balancing security and efficiency. In addition, there are also studies attempting to combine national security algorithms with emerging security technologies. Liu et al. explored an IoT authentication scheme based on SM9 (Identity Cryptography Algorithm) [15], but SM9 also faces the issue of key custody.

However, the uncertified cryptographic system still relies on secure storage of private keys. Once an IoT terminal is physically obtained by an attacker, its internal stored private key may be extracted or cloned through techniques such as side channel attacks and microprobes, leading to the collapse of the entire authentication system. Physical unclonable function (PUF) technology provides a new approach for solving this fundamental problem [16]. PUF utilizes the inherent and uncontrollable microphysical differences in the semiconductor manufacturing process, such as gate delay, wiring delay, etc., to generate a unique “digital fingerprint” of the device. This response has the characteristics of unpredictability, unclonability, and tamper resistance, and it does not require permanent storage of keys in non-volatile memory, achieving “implicit” security [17]. By combining PUF with cryptographic primitives, a security barrier rooted in the hardware itself can be constructed [18].

In view of this, this article proposes an IoT terminal identity authentication mechanism that integrates the SM2 uncertified cryptographic system and PUF technology. This mechanism aims to fully leverage the advantages of both parties: using the SM2 certificateless system to eliminate certificate management overhead and key custody issues and achieving efficient and compliant identity authentication; using PUF technology to generate unique identity identifiers and key materials for terminal devices and seamlessly embedding them into the uncertified private key generation process so that the final generated private key is physically bound to the terminal hardware. Even if an attacker steals some system data, they cannot replicate or calculate the private key on another device, effectively resisting physical and cloning attacks from the source.

In summary, existing certificateless authentication schemes mainly focus on the security of cryptographic protocols, lacking effective responses to physical security threats to terminals, and the problem of private key storage has not been eradicated. Although there have been studies combining PUF with cryptography, most have focused on IBC or traditional international algorithms, and there are still few studies that deeply and consistently integrate PUF with SM2 certificateless systems. Therefore, this article aims to bridge the above gap and propose an innovative IoT terminal identity authentication mechanism that deeply integrates the SM2 uncertified cryptographic system and PUF technology in order to achieve higher levels of security while ensuring lightweight efficiency.

The main work and contributions of this article are as follows:

- Analyze the security challenges faced by IoT terminal identity authentication and the limitations of existing solutions.
- Design a new authentication architecture that uses PUF as the hardware root of trust and deeply integrates it with SM2 uncertified cryptography.
- Elaborate on the initialization, key generation, registration, and authentication protocol process of the mechanism.
- Analyze and evaluate the proposed mechanism from the perspectives of security and performance and demonstrate its ability to effectively resist various known attacks while also being suitable for resource-constrained IoT environments in terms of computational and communication overhead.

## 2. Background

### 2.1. Certificateless Public Key Mechanisms

The Certificateless Cryptography System (CL-PKC) was first proposed by Al Riyami and Paterson in 2003 [11], with the original intention of finding a balance between avoiding the certificate management complexity of traditional Public Key Infrastructure (PKI) and the key custody problem based on Identity-Based Cryptography (IBC). Once proposed, this system became a research hotspot in the fields of cryptography and security.

In terms of authentication and key negotiation, numerous scholars have conducted in-depth explorations of uncertified authentication protocols. He et al. proposed an efficient certificateless authentication protocol [19] but later pointed out that it is susceptible to temporary key leakage attacks and key replacement attacks. To enhance security, subsequent studies, such as Islam et al., have introduced more complex bidirectional authentication mechanisms [20] but often at the cost of increased computational and communication overhead. Most of these studies are based on traditional algorithms such as RSA or DH, while certificateless schemes based on elliptic curves are more favored in resource-constrained environments due to their higher efficiency. The SM2 algorithm, as the standard for elliptic curves in China, is of great significance for its undocumented application research. Peng et al. proposed the application of SM2 in a certificateless system [21], but their solution did not fully consider the physical security threats of IoT terminals and lacked deep integration with hardware security technology. The SM2 certificateless signature mechanism is a signature scheme based on the certificateless cryptographic system (CL-PKC), and its detailed algorithm includes the following content:

- CL.Setup ($1^{k}$): Given the security parameter k, this function initializes the uncertified password system and generates the system’s master public key and master private key (Mpk;Msk). This function is executed by KGC.
- CL.Set-User-Key (Mpk): This function generates the user’s partial public key and partial private key ($U_{A};x_{A}$).
- CL.Extract-Partial-Key ($Mpk,Msk,{\mathrm{ID}}_{A},U_{A}$): This function is executed by KGC and generates the parts for the users’ public key and partial private key ($W_{A}$; $d_{A}$).
- CL.Set-Public-Key (Mpk, $ID_{A}$, $U_{A}$, $W_{A}$): This function generates the public key $P_{A}$ (implicit certificate password) declared by the user, also known as public key restoration data in the system.
- CL.Encrypt (Mpk, $ID_{A}$, $P_{A}$, m): This function encrypts message m to generate ciphertext C.
- CL.Decrypt (Mpk, $ID_{A}$, $P_{A}$, $s_{A}$, C): This function decrypts ciphertext C and outputs m or a terminator ⊥.

### 2.2. PUF Technology

Physical Unclonable Function (PUF) is a technique that utilizes microscopic physical differences generated during the hardware manufacturing process to generate a unique “fingerprint” of a device. Due to its randomness, uniqueness, and unclonability in output, PUF is known as the “hardware trust root” and provides a novel solution for secure authentication [22].

Early research mainly utilized the response generated by PUF to directly generate keys or serve as credentials for challenge–response authentication. Gassend et al. first used PUF for device identification [23]. Guajardo et al. proposed using PUFs to securely generate and store cryptographic keys [24], avoiding the problem of non-volatile storage of keys. However, the simple challenge response mode may appear fragile in the face of modeling attacks.

To enhance robustness and expand application scenarios, the research trend is shifting towards combining PUF with advanced cryptographic protocols. Sadeghi et al. proposed the concept of “PUF-based Byzantine Agreement” and used PUF for lightweight authentication [25]. Kulseng et al. proposed a lightweight authentication protocol based on PUF [26], but this scheme does not use public key cryptography and has poor scalability. A more valuable research direction is the work of Chatterjee et al., who combined PUF responses as part of user private keys with the IBC system, effectively alleviating the key custody problem of IBC [27]. This provides important insights for this study to combine PUF with certificateless systems. However, existing integration schemes are mostly based on international standard algorithms such as ECDSA and RSA, and research on the authentication mechanism for the deep integration of China’s independent SM2 algorithm and PUF is not yet sufficient.

SRAM-PUF, in particular, has been applied to large-scale industrialization due to its flexibility, practicality, and low cost. However, Helfmeier et al. [28] argue that SRAM-PPU is susceptible to physical cloning due to its compact structure and robustness to environmental impacts. They also pointed out that physical cloning takes over 20 h to complete and is extremely costly. On the other hand, some high-security PUFs may have drawbacks, such as high consumption, requiring additional hardware, or affecting the storage itself. However, in recent years, many researchers have proposed various solutions against anti-physical cloning attacks [17,29]. For practical purposes, we take SRAM-PUF as an example in the proposed PUF-SM2 authentication scheme, and its actual application process can be summarized as the following two steps:

- PUF Security Chip Registration Stage:

This occurs in a controlled and secure environment, such as chip production testing or first-time configuration. At this point, multiple challenges $\;C_{i}$ are applied to the PUF security chip, and raw responses $R_{i}$ are generated for each challenge. Finally, the most reliable and noise-free feature values are estimated by taking the average of multiple measurements. Then, use error correction codes (BCH codes, RS codes, or dedicated schemes) to generate auxiliary data. This is not a secret, and it can be stored publicly. This process contains sufficient information to correct bit errors within the expected range but does not disclose any substantive information about the response R and final key K. Then, apply a cryptographically secure pseudo-random function (such as the cryptographic hash function SHA-256) to R or part of it to generate a stable high-entropy root key  K=Hash(R). Ultimately, only auxiliary data needs to be stored.

- Key Reconstruction/Verification Phase:

This process is only carried out when a key is needed during the daily operation of the device. Firstly, input the challenge, where the PUF security chip generates a noisy on-site response $R^{\prime }$. Using stored auxiliary data P  and noisy on-site response $R^{\prime }$, run the recovery algorithm of the fuzzy extractor again to ultimately reconstruct the same key K.

## 3. System Model and Security Model

### 3.1. System Model

**Figure 1:**
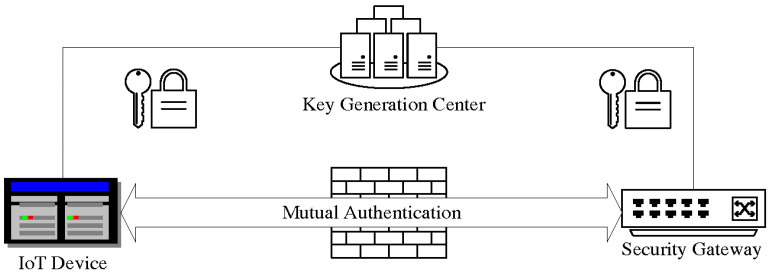
The system model.

Our system mainly consists of three main components, namely the digital identity authentication center, IoT terminal devices, and the security gateway. The model of the entire system is shown in Figure 1.

The Digital Identity Certification Center (KGC) is a trusted entity in the uncertified cryptographic system, but its level of trustworthiness is much lower than that of the Certificate Authority (CA) in traditional Public Key Infrastructure (PKI). KGC is responsible for generating “partial private keys” for system users, but it does not know the user’s complete private key. This is the most critical feature of certificateless cryptography, which solves the certificate management problem of traditional PKI and the key escrow problem rooted in Identity-Based Cryptography (IBC).

IoT terminal devices are composed of various embedded devices with highly limited resources. They are responsible for collecting data and executing control instructions. The biggest difference between our system’s terminal devices and traditional IoT devices is the loading of PUF security chips.

The security gateway, as an intermediate node connecting the past and the future, aggregates data from multiple terminal devices and communicates with the application platform through high-speed networks such as Ethernet and 4G/5G. Gateway usually has strong computing and storage capabilities, which can assist in performing some security functions. The identity of IoT terminal devices attempting access is verified, ensuring that only legitimate devices can access the system and participate in data exchange.

### 3.2. Security Model

Traditional certificateless public key cryptography is subject to two types of attacks: one from external attackers (who are malicious users) and the other from internal attackers (who are malicious KGCs). Similarly, based on the signature schemes with formal security proofs [30], our scheme also faces two PPT adversaries: Type I adversary $A_{1}$ and Type II adversary $A_{1}$. Here, $A_{1}$ is a malicious user who can replace any user’s public key but does not know the master key; $A_{2}$ is a malicious KGC who has the master key but cannot replace any user’s public key. Specifically, $A_{1}$ cannot replace the public key of the challenged identity before the challenge phase, and $A_{2}$ does not need to perform partial private key generation queries as they can calculate them independently. Moreover, this paper further enriches the attack capabilities of the adversaries. Besides the two attack capabilities described above, the adversaries discussed in this paper can also physically attack the devices to obtain all stored public or secret parameters.

## 4. PUF + SM2 Certificateless Identity Authentication Mechanism

The PUF + SM2 solution we propose aims to address the challenge of how to securely generate and store keys and conduct mutual authentication for IoT devices in resource-constrained environments. It utilizes hardware fingerprints (PUF) as the trust root and combines the high security of the national cryptographic SM2 algorithm to build a full-process security loop from device initialization to cloud authentication. The first stage is system initialization (System Initialization), where the KGC defines system parameters and publicly discloses parameters to issue corresponding authentication policies for terminals. The second stage is terminal private key setting (Terminal Private Key Setting), which occurs when IoT terminals are produced or first started. The core of this stage is to generate a “fingerprint” based on the physical characteristics of the terminal chip instead of traditionally storing keys. The third stage is KGC setting private keys (KGC Setting Private Keys), which refers to the KGC generating another partial public–private key pair associated with the submitted partial public key when it receives a key application from the terminal. The fourth stage is mutual authentication (Mutual Authentication), where, based on the certificateless signature protocol, the terminal and the security gateway achieve mutual authentication. The fifth stage is signed enveloped data (Signed Enveloped Data), which, on the basis of mutual authentication, builds a signed data transmission protocol that not only protects the confidentiality of the data but also ensures its integrity.

### 4.1. System Initialization

The digital identity authentication center invokes the SM2 certificateless initialization algorithm and obtains a set of system parameters as follows:

$$SP=\{G,p,a,b,n,G,P_{pub},H_{1},H_{2},H_{3}\}$$

Among them, there is a cyclic group G based on parameter p,a,and b′s customized elliptic curves, and the order of this group is n, with G as one of its generators. According to the national security GM/T 0005 standard [31], select a true random number $mk\in \left[1,n-1\right]$ and calculate the system’s master public key $P_{pub}=\left[mk\right]G$. Then, select three hash functions $\left\{H_{1},H_{2},H_{3}\right\}$ that meet the national security SM3 algorithm.

### 4.2. Terminal Private Key Setting

The IoT terminal ${ID}_{Device}$ loaded with the PUF security chip reads the characteristic values of SRAM PUF $R^{\prime }$ in a controlled environment. Error-correcting codes use stored auxiliary data P to correct erroneous bits, successfully reconstructing the original feature values R and applying the same cryptographic hash function again. The IoT node generates a unique identity identifier ${UID}_{Device}$ based on the root key  K=Hash(R) generated by the physical characteristics of the PUF chip, as well as identity information $\;{ID}_{Device}$. This identity identifier cannot be forged or tampered with. Initiate a registration application to the digital identity authentication platform; first, generate a root key uniquely associated with the chip fingerprint based on the PUF features, derive a key $x_{Device}\in \left[1,n-1\right]$ from the root key K as terminal private key component one, and calculate public key component one, $X_{Device}=[x_{Device}]G$. Then, the terminal will send it together to the digital identity authentication center for registration and key application.

Similarly, the gateway generates a unique identity identifier ${UID}_{GW}$ based on the root key generated by PUF physical characteristics and identity information tagging, which cannot be forged or tampered with. Initiate a registration application to the digital identity authentication platform, and derive a random number of keys $x_{GW}\in \left[1,n-1\right]$ based on the root key generated by the PUF chip as terminal private key component one. The security gateway calculates the public key component $X_{GW}=[x_{GW}]G$. Then, the security gateway will send it to the digital identity authentication center for registration and key application.

### 4.3. KGC Setting Private Keys

For IoT terminals, the digital identity authentication platform first registers ${UID}_{Device}$ and configures authentication policies and permission control policies for its terminals. The digital identity authentication center generates public and private key component two for the terminal based on the system’s master key and public parameters. The specific generation method is as follows:

$$Z_{Device}=H_{1}\left(\begin{array}{c} UID_{Device}∥a∥b∥x_{G}∥y_{G}∥x_{P_{Pub}}∥y_{P_{Pub}} \end{array}\right)$$

$$w_{Device}\in \left[1,n-1\right]$$

$$W_{Device}=\left[w_{Device}\right]G+X_{Device}$$

$$\lambda_{Device}=H_{2}\left(x_{W_{Device}}∥y_{W_{Device}}∥Z_{Device}\right)$$

$$t_{Device}=\left(w_{Device}+\lambda_{Device}\cdot mk\right)\;mod\;$$

The final output of public key component $W_{Device}$ and private key component $t_{Device}$ is securely sent to the terminal ${UID}_{Device}$.

It should be emphasized here that, unlike traditional uncertified public key mechanisms, private key component two is no longer stored locally in plaintext but is transmitted and stored in ciphertext. Our PUF + SM2 certificateless bidirectional authentication mechanism adopts the most direct implementation method, applying traditional ElGamal encryption methods to encrypt the public key component of the application terminal, saving it as ${ct}_{Device}^{*}$, and then publicly disclosing the terminal’s public key.

Similarly, for security gateways, the digital identity authentication center distributes terminal authentication policies and permission control policies to the gateway. The digital identity authentication center generates public and private key component two for the gateway based on the system’s master key and public parameters. The specific generation method is as follows:

$$Z_{GW}=H_{1}\left(\begin{array}{c} UID_{GW}∥a∥b∥x_{G}∥y_{G}∥x_{P_{Pub}}∥y_{P_{Pub}} \end{array}\right)$$

$$W_{GW}=\left[w_{GW}\right]G+X_{GW}$$

$$\lambda_{GW}=H_{2}\left(x_{W_{GW}}∥y_{W_{GW}}∥Z_{GW}\right)$$

$$t_{GW}=\left(w_{GW}+\lambda_{GW}\cdot mk\right)\;mod\;n$$

Ultimately, it is also transmitted and stored in encrypted form ${ct}_{GW}^{*}$, encrypting the public key component of the application security gateway and storing it as the public key of the terminal, which is then made public.

### 4.4. Mutual Authentication

The terminal device generates a signature about the current timestamp based on its own private key and sends it to the security gateway for signature verification. The security gateway calculates the complete public key based on the terminal’s public key and performs digital signature verification.

Firstly, the IoT terminal retrieves key component one, $x_{Device}$, based on the physical characteristics of PUF, decrypts the ciphertext ${ct}_{Device}^{*}$ to obtain key component $t_{Device}$, and reconstructs its complete private key, ${sk}_{Device}=\left(x_{Device}+t_{Device}\right)mod\;n$.

Then, according to the signature algorithm, perform a signature operation on the timestamp Γ. Randomly select $\in \left[1,n-1\right]$, and the signature $\sigma =\left(u,v\right)$ can be computed as follows:

$$Z_{Device}=H_{1}\left(\begin{array}{c} UID_{Device}∥a∥b∥x_{G}∥y_{G}∥x_{P_{Pub}}∥y_{P_{Pub}} \end{array}\right)$$

$$\lambda_{Device}=H_{2}\left(x_{W_{Device}}∥y_{W_{Device}}∥Z_{Device}\right)$$

$$h=H_{3}(\lambda_{Device}∥\Gamma )$$

$$Q=\left[r\right]G$$

$$u=\left[x_{Q}\right]\;mod\;n$$

$$v=r^{-1}\cdot \left(u\cdot sk_{Device}+h\right)\;mod\;n$$

Then, send an authentication request to the security gateway, which includes ${UID}_{Device},\;W_{Device},\;\Gamma$, and signature information σ.

The security gateway calculates the complete public key of the terminal based on the public key of the digital identity authentication center and the public parameters of the system. The process is as follows:

$$Z_{Device}=H_{1}\left(\begin{array}{c} UID_{Device}∥a∥b∥x_{G}∥y_{G}∥x_{P_{Pub}}∥y_{P_{Pub}} \end{array}\right)$$

$$\lambda_{Device}=H_{2}\left(x_{W_{Device}}∥y_{W_{Device}}∥Z_{Device}\right)$$

$$pk_{Device}=\left[W_{Device}\right]G+\left[\lambda_{Device}\right]P_{Pub}$$

$$h=H_{3}(\lambda_{Device}||\Gamma )$$

$$v_{1}=v^{-1}\cdot h\;mod\;n$$

$$v_{2}=v^{-1}\cdot u\;mod\;n$$

$$Q^{\prime }=\left[v_{1}\right]G+\left[v_{2}\right]pk_{Device}$$

$$u^{\prime }=x_{Q^{\prime }}\;mod\;n$$

If $u^{\prime }=u\;$ holds, the verification is passed. Otherwise, the verification will be invalid.

Similarly, the security gateway retrieves key component one, $x_{GW}$, based on the physical characteristics of PUF, decrypts the ciphertext ${ct}_{GW}^{*}$ to obtain key component $t_{GW}$, and reconstructs its complete private key, ${sk}_{GW}=\left(x_{GW}+t_{GW}\right)mod\;n$.

Then, according to the signature algorithm in the uncertified standard, perform a signature operation on the timestamp $\Gamma^{*}$. Randomly select $r^{*}\in \left[1,n-1\right]$, and the signature $\sigma^{*}=\left(u^{*},v^{*}\right)$ can be computed as follows:

$$Z_{GW}=H_{1}\left(\begin{array}{c} UID_{GW}∥a∥b∥x_{G}∥y_{G}∥x_{P_{Pub}}∥y_{P_{Pub}} \end{array}\right)$$

$$\lambda_{GW}=H_{2}\left(x_{W_{GW}}∥y_{W_{GW}}∥Z_{GW}\right)$$

$$h^{*}=H_{3}(\lambda_{GW}∥\Gamma^{*})$$

$$Q^{*}=\left[r^{*}\right]G$$

$$u^{*}=\left[x_{Q^{*}}\right]\;mod\;n$$

$$v^{*}={r^{*}}^{-1}\cdot \left(u^{*}\cdot sk_{GW}+h^{*}\right)\;mod\;n$$

Then, send an authentication request to the security gateway, which includes ${UID}_{GW},\;W_{GW},\;\Gamma^{*}$, and signature information $\sigma^{*}$.

The terminal calculates the complete public key of the security gateway based on the public key of the digital identity authentication center and the public parameters of the system. The process is as follows:

$$Z_{GW}=H_{1}\left(\begin{array}{c} UID_{GW}∥a∥b∥x_{G}∥y_{G}∥x_{P_{Pub}}∥y_{P_{Pub}} \end{array}\right)$$

$$\lambda_{GW}=H_{2}\left(x_{W_{GW}}∥y_{W_{GW}}∥Z_{GW}\right)$$

$$pk_{GW}=\left[W_{GW}\right]G+\left[\lambda_{GW}\right]P_{Pub}$$

$$h^{*}=H_{3}(\lambda_{GW}∥\Gamma^{*})$$

$$v_{1}^{*}={v^{*}}^{-1}\cdot h^{*}\;mod\;n$$

$$v_{2}^{*}={v^{*}}^{-1}\cdot u^{*}\;mod\;n$$

$${Q^{*}}^{\prime }=\left[v_{1}^{*}\right]G+\left[v_{2}^{*}\right]pk_{GW}$$

$${u^{*}}^{\prime }=x_{{Q^{*}}^{\prime }}\;mod\;n$$

If ${u^{*}}^{\prime }=u^{*}\;$ holds, the verification is passed. Otherwise, the verification will be invalid.

### 4.5. Signed Enveloped Data

**Figure 2:**
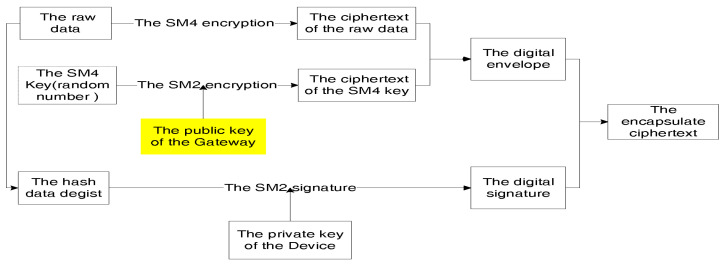
The encryption processes of SED.

**Figure 3:**
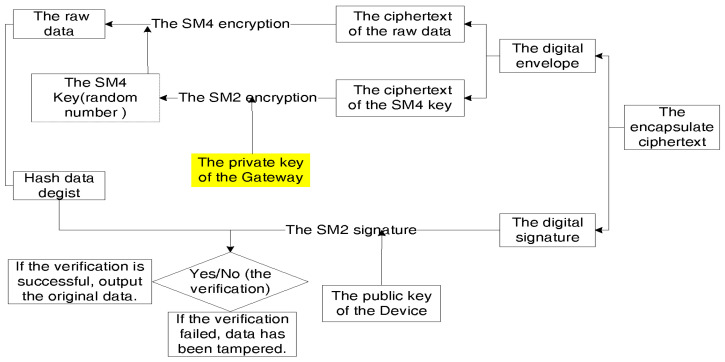
The decryption processes of SED.

Finally, we applied the authentication scheme above and designed a lightweight encrypted communication protocol called SED. Taking the example of the terminal sending information to the gateway, we will describe the encryption and decryption processes using Figure 2 and Figure 3, respectively.

## 5. Discussion

### 5.1. Security Analysis

#### 5.1.1. The Formal Security Proof

On the basis of the new security model, the formal security proof of our scheme’s certificateless public key signature protocol has been rigorously described in relevant schemes [30]. The fundamental difference between our solution and others is that the function of PUF is to generate a private key associated with the hardware, which replaces the randomly selected user private key x in the CL.Set User Key algorithm. In general, this is usually injected by a third party and not bound to the identity of the hardware, so there is a threat of physical attacks. Our application of PUF technology has fundamentally changed the way this key is generated, from external generation to internal self-generation. But fundamentally, it does not change the security of our lack of certificates.

#### 5.1.2. Signature Non-Forgeability

Under the assumption of the random oracle model and computational difficulty and based on the formal security analysis of the existing SM2 signature algorithm [30], our PUF + SM2 certificateless bidirectional identity authentication scheme can also resist both Type I and Type II attacks, successfully achieving the non-forgeability of signatures. I will not go into detail here.

#### 5.1.3. Identity Authentication

Our scheme’s identity authentication is protected by the immutability of the SM2 certificateless signature algorithm. We know that the authentication messages in the above identity authentication protocol are unforgeable, which means that, in our mechanism, no adversary can forge valid request and response messages. In this case, if the transmitted message is verified, this means that the corresponding sender has a legitimate identity.

#### 5.1.4. Data Integrity

In our PUF + SM2 authentication scheme, after successful authentication, the encryption key is negotiated. Data integrity refers to ensuring that data is not modified or tampered with during transmission or storage. In our mechanism, the message adopts a signed enveloped data mode. If the data is tampered with, the data summary will definitely change, ensuring the integrity of the data.

#### 5.1.5. Resist Physical Attacks

In our PUF + SM2 certificateless bidirectional authentication mechanism, a unique identity identifier (UID) is derived based on the physical characteristics of PUF. The terminal device does not need to explicitly store the key in its local memory. Therefore, the proposed solution can safely withstand physical attacks.

### 5.2. Performance Analysis

The security of our PUF + SM2 certificateless bidirectional authentication mechanism has been demonstrated, so in this section, we will analyze the performance of our solution, such as computational efficiency and communication efficiency. It should be noted that communication efficiency is determined by the length of the signature. We first compare the proposed PUF + SM2 scheme with related schemes, including existing uncertified signature schemes. Of course, we have not listed all uncertified schemes. On the contrary, only some commonly cited pairing-based schemes [12,32] and some of the most effective unpaired schemes [33] are compared.

Firstly, the basic algorithms for low-level computation are shown in Table 1. In order to further demonstrate the comparison of computational costs and communication efficiency between different structures, the comparison results of signature lengths are shown in Table 2. At the same time, we simulated the basic underlying operations mentioned in our solution and the comparative solution for a common hardware environment, finally presenting Table 3, which includes a comparison of our computation time. At the same time, quantitative statistics were conducted for the efficiency comparison of storage overhead and signature length.

## 6. Conclusions

This article focuses on the security challenges of limited resources and susceptibility to physical theft and cloning attacks in IoT terminal devices. It deeply studies existing identity authentication mechanisms and proposes an IoT terminal identity authentication scheme that integrates the national security SM2 uncertified public key cryptosystem with physically unclonable functions (PUFs). This article successfully designs and implements a secure and efficient authentication model. The core innovation of this model lies in utilizing the physical unclonability of PUF to deeply bind the identity of the terminal device to its hardware itself. The unique response generated by PUF is used as a seed to generate the user’s partial private key, fundamentally eliminating the possibility of attackers cloning devices by copying keys or software images and providing the first layer of hardware-level security for the system. At the same time, the model adopts a certificateless cryptographic framework, which completely eliminates the high cost of certificate management, storage, and verification in traditional PKI systems, and it also avoids the inherent key custody problem in identity-based cryptographic systems, achieving a good balance between security and efficiency. Secondly, the comprehensive advantages of the proposed scheme were verified through rigorous formal security analysis and performance evaluation. In terms of security, the scheme has been proven to be secure under the random oracle model, effectively resisting various security threats such as physical cloning attacks, man-in-the-middle attacks, replay attacks, and meeting security goals such as two-way authentication, session key negotiation, and forward security. In terms of performance, the scheme cleverly allocates the computationally intensive SM2 public key operations mainly to resource-rich server-side processing, greatly reducing the computational and storage burden on terminal nodes, making it very suitable for IoT terminal environments with extremely limited computing power, storage space, and energy.

## Figures and Tables

**Table 1:** Symbol of the mathematical operations.

Symbol	The Mathematical Operations
$T_{rc}$	Modular inversion computation
$T_{meo}$	Modular exponentiation operation
$T_{smec}$	Scalar multiplication on elliptic curve
$T_{paec}$	Point addition on elliptic curve

**Table 2:** Qualitative comparison results of different mechanisms.

The Scheme	Signature	Verification	The Type of the Adversary	Physical Attack
[12]	$1T_{smec}+1T_{rc}$	$4T_{smec}+2T_{paec}$	Not secure	No
[32]	$1T_{smec}+1T_{rc}$	$4T_{smec}+2T_{paec}$	Type II	No
[33]	1$T_{meo}$	4$T_{meo}$	Type I & II	No
SM2	1$T_{smec}$	2$T_{smec}$ + 1$T_{paec}$	Type I & II	No
PUF + SM2	1$T_{smec}$	2$T_{smec}$ + 1$T_{paec}$	Type I & II	Yes

**Table 3:** Quantitative comparison results of different mechanisms.

The Scheme	The Cost of Signature (ms)	The Cost of Verification (ms)	The Signature Size (Bits)
[12]	11.7723	40.337	480
[32]	11.7723	40.337	480
[33]	16.7161	66.8644	1184
SM2	10.0426	20.1685	320
PUF + SM2	10.0426	20.1685	320

## Data Availability

The original contributions presented in this study are included in the article; further inquiries can be directed to the corresponding author.

## Abbreviations

The following abbreviations are used in this manuscript:

PUF	Physical Unclonable Function
CL-PKC	Certificateless Public Key Cryptography
IBC	Identity-Based Cryptography
ECC	Elliptic Curve Cryptography
KGC	Key Generation Center
SED	Signed Enveloped Data
